# Growth hormone deficiency testing and treatment following mild traumatic brain injury

**DOI:** 10.1038/s41598-021-87385-7

**Published:** 2021-04-20

**Authors:** Leah J. Mercier, Natalia Kruger, Quynk B. Le, Tak S. Fung, Gregory A. Kline, Chantel T. Debert

**Affiliations:** 1 Division of Physical Medicine and Rehabilitation, Department of Clinical Neurosciences, University of Calgary, Calgary, AB Canada; 2 Endocrinology and Metabolism Program, Alberta Health Services, Calgary, AB Canada; 3 Faculty of Nursing, University of Calgary, Calgary, AB Canada; 4 Division of Endocrinology, Department of Medicine, University of Calgary, Calgary, AB Canada

**Keywords:** Brain injuries, Pituitary diseases, Rehabilitation

## Abstract

Pituitary dysfunction, specifically growth hormone (GH) deficiency, can occur following traumatic brain injury. Our objective was to characterize the prevalence of GH deficiency (GHD) testing and response to recombinant human GH (rhGH) treatment in adults with persistent symptoms following mild traumatic brain injury (mTBI) referred for assessment of pituitary dysfunction. A retrospective chart review was conducted of patients seen at an outpatient brain injury clinic with a diagnosis of mTBI and persistent post-concussive symptoms who were referred to endocrinology. Clinical assessments of symptoms were collected. Investigations and results of GHD were collected, including initiation of rhGH treatment and treatment response. Of the 253 patients seen in both brain injury and endocrinology clinics, 97 with mTBI were referred for investigation of pituitary dysfunction and 73 (75%) had dynamic testing for assessment of GHD. Of the 26 individuals diagnosed with GHD, 23 (88%) started rhGH. GH therapy was inconsistently offered based on interpretation of GH dynamic testing results. Of those who started rhGH, 18 (78%) had a useful treatment response. This study suggests that clinical management of these patients is varied, highlighting a need for clear guidelines for the diagnosis and management of GHD following mTBI.

## Introduction

In Canada, the estimated annual incidence of mild traumatic brain injury (mTBI) is 1153/100,000^1^, posing considerable public health challenges. While the majority of individuals have symptom resolution in the 10–14 day period following mTBI, up to 30% struggle with symptoms greater than 3 months post-injury, defined as persistent post-concussive symptoms^2^. Persistent symptoms are characterized by headache, dizziness, fatigue, cognitive deficits and sleep disturbances, among other chronic impairments^2^. It has long been recognized that traumatic brain injury (TBI) can result in pituitary dysfunction^3^, however a meta-analysis of post-traumatic anterior pituitary dysfunction reported that only 16.4% of included participants had an mTBI^4^. At one-year post-mTBI the risk of growth hormone (GH) deficiency ranges from 5 to 40%^5–8^ depending on disease definition and mode of investigation. Incidence data are from small cohorts of nearly exclusively hospitalized patients, which does not represent the majority of individuals who sustain an mTBI.

Expert opinions have suggested neuroendocrine screening should be offered to mTBI patients requiring hospitalization for at least 24 h or those with complicated mTBI, defined as incranial bleed, brain swelling or skull fracture on initial computerized tomography (CT) scan^9^. However, neuroendocrine deficits can occur in patients with mTBI whether an abnormality on CT is present or not^10^. Preliminary studies have suggested benefits of treating post-traumatic GH deficiency (GHD) in patients with mTBI, as well as those following moderate to severe TBI^11–13^. Therefore, the current study had two objectives: (1) characterize GHD testing and treatment response in an adult cohort following mTBI; (2) evaluate associations between clinical outcomes and GHD.

## Results

### Patient referral to outpatient endocrinology clinics (OECs)

**Figure 1:**
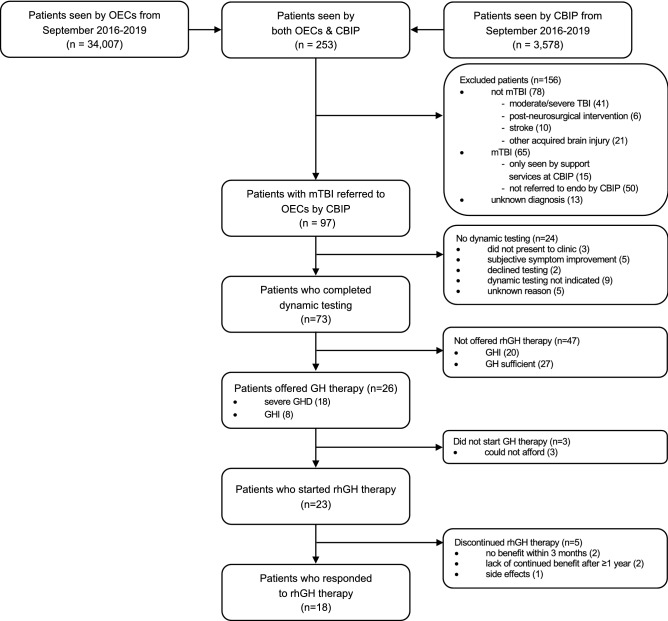
Flowchart of referral to endocrinology, GH testing and rhGH therapy. *CBIP* Calgary Brain Injury Program (outpatient brain injury clinic), *GH* growth hormone, *GHD* growth hormone deficiency, *GHI* growth hormone insufficiency, *mTBI* mild traumatic brain injury, *OECs* outpatient endocrinology clinics, *rhGH* recombinant human growth hormone, *TBI* traumatic brain injury.

Of the 37,585 patients seen in the outpatient brain injury clinic (CBIP) and outpatient endocrinology clinics (OECs) during the study period, 253 patients were common between the two services. Of the patients seen by both services, 97 were referred to OECs by a CBIP physician for investigation of post-traumatic pituitary dysfunction. The excluded patients either did not sustain an mTBI (n = 78), were only seen by support services at CBIP (n = 15) or were referred to OECs by another treating physician (n = 50). A diagnosis could not be confirmed in the case of 13 charts and therefore were excluded (Fig. 1). Patient characteristics and past medical history of referred patients and those who underwent dynamic testing are reported in Table 1.

**Table 1:** Demographics and past medical history of patients referred to OECs and who completed dynamic testing.

	Patients referred to OECs (n = 97)	Patients who completed dynamic testing (n = 73)
**Demographics**		
Age at injury, n, M (SD)	91, 40.0 (12.6)	68, 42.3 (11.6)
**Sex, n (%)**		
Female	52 (53.6)	40 (54.8)
Male	45 (46.4)	33 (45.2)
**Handedness, n (%)**		
Right	87 (92.6)	66 (94.3)
Left	7 (7.4)	4 (5.7)
**Education, n (%)**		
High school or less	17 (19.1)	7 (10.6)
Technical degree/vocational	16 (18.0)	11 (16.7)
Bachelor’s degree	37 (41.6)	30 (45.5)
Graduate/professional degree	19 (21.3)	18 (27.3)
**Employment, n (%)**		
Full time	29 (31.5)	22 (32.4)
Part time	13 (14.1)	10 (14.7)
Disability	19 (20.7)	16 (23.5)
Student	6 (6.5)	2 (2.9)
Not working	25 (27.2)	18 (26.5)
**Injury characteristics**		
**Radiographic finding of complicated mTBI, n (%)**		
Yes	3 (3.1)	3 (4.1)
No	94 (96.9)	70 (95.9)
**Past medical history**		
**Previous mTBI, n (%)**		
Yes	44 (45.4)	34 (46.6)
No	53 (54.6)	39 (53.4)
**Endocrine disorder, n (%)**		
One or more	23 (23.7)	17 (23.3)
Hypercholesterolemia	2 (2.1)	2 (2.7)
Diabetes/prediabetes	7 (7.2)	3 (4.1)
Hypogonadism	1 (1.0)	0 (0.0)
Hypothyroidism	7 (7.2)	7 (9.6)
Galactorrhea	2 (2.1)	1 (1.4)
Dyslipidemia	8 (8.2)	6 (8.2)
Hashimoto thyroiditis	2 (2.1)	2 (2.7)

*mTBI* mild traumatic brain injury, *OECs* outpatient endocrinology clinics.

### Growth hormone (GH) testing

Of the 97 CBIP-assessed patients referred to OECs, 73 (75%) completed dynamic testing. Patients did not receive testing for the following reasons: 3 did not present to clinic, 5 patients reported subjective improvement of post-concussive symptoms, 2 declined testing and for 9 patients the treating endocrinologist reported dynamic testing was not indicated. Of those 9 patients, 3 had serum insulin-like growth factor 1 (IGF-1) as the only investigation of GHD. In the case of 5 patients, reason for lack of dynamic testing could not be determined from electronic medical records (Fig. 1). Patients who completed dynamic testing had a mean age of 43.9 ± 11.8 years at time of testing with 55% being female. Individuals were a mean of 24.8 ± 11.3 months post-mTBI.

**Figure 2:**
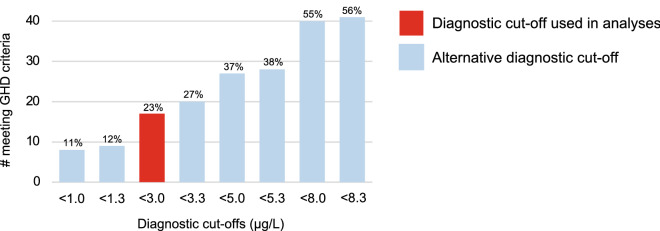
GHD prevalence using alternative diagnostic cut-offs based on peak GH during dynamic testing. Numbers of patients meeting differential diagnostic cut-offs with percentages calculated out of total number of individuals tested (n = 73). *GH* growth hormone, *GHD* growth hormone deficiency.

Only 3 patients (4%) who completed testing had a complicated mTBI, defined as presence of intracranial bleed or contusion on imaging. The majority (94%) of patients underwent a glucagon stimulation test (GST), while 6% had an insulin tolerance test (ITT). Of the 73 patients who completed dynamic testing 17 (23%) had severe GHD and 29 (40%) had GH insufficiency (GHI). Two patients with severe GHD also had post-traumatic hypogonadism. None of the patients with severe GHD had a complicated mTBI. Across groups there were no differences in age at testing, sex, time since injury, mechanism of injury, loss of consciousness at injury or post-traumatic amnesia (Table 2). Sensitivity analysis for GHD prevalence using different peak GH cut-offs for GHD is presented in Fig. 2.

**Table 2:** Demographics, injury characteristics and endocrine assessment results according to dynamic testing results and treatment response.

	Completed dynamic testing (n = 73)				Started rhGH treatment (n = 23)		
	Severe GHD (n = 17)	GHI (n = 29)	GH sufficient (n = 27)	p value	Responders (n = 18)	Non-responders (n = 5)	p value
**Demographics**							
Age at dynamic testing, n, M (SD)	17, 45.2 (10.1)	29, 45.8 (13.5)	26, 41.0 (10.4)	0.276	18, 43.4 (10.6)	5, 57.6 (7.8)	0.012*
**Sex, n (%)**				$\chi$ ^2^(2,N = 73) = 3.508, p = 0.194			$\chi$ ^2^(1,N = 23) = 1.791, p = 0.297
Female	6 (35.3)	17 (58.6)	17 (63.0)		5 (27.8)	3 (60.0)	
Male	11 (64.7)	12 (41.4)	10 (37.0)		13 (72.2)	2 (40.0)	
**Injury characteristics**							
Months since injury at CBIP clinic visit, n, M (SD)	16, 13.5 (10.2)	24, 9.3 (6.7)	22, 9.0 (9.8)	0.246	15, 12.7 (9.3)	5, 11.6 (6.2)	0.816
Months since injury at dynamic testing, n, M (SD)	16, 24.2 (12.2)	24, 25.0 (11.9)	22, 25.1 (10.5)	0.966	15, 22.8 (11.7)	5, 25.6 (11.4)	0.646
**Radiographic finding of complicated mTBI, n (%)**				$\chi$ ^2^(2,N = 73) = 1.506, p = 0.610			
Complicated mTBI	0 (0.0)	1 (3.4)	2 (7.4)		0 (0.0)	0 (0.0)	
Uncomplicated mTBI	17 (100.0)	28 (96.6)	25 (92.6)		18 (100.0)	5 (100.0)	
**Mechanism of injury, n (%)**				$\chi$ ^2^(8,N = 73) = 9.878, p = 0.277			$\chi$ ^2^(3,N = 23) = 4.779, p = 0.230
Sports/recreation	5 (29.4)	14 (48.3)	9 (33.3)		8 (44.4)	0 (0.0)	
Fall	3 (17.6)	3 (10.3)	4 (14.8)		2 (11.1)	2 (40.0)	
Motor vehicle collision	8 (47.1)	10 (34.5)	8 (29.6)		7 (38.9)	3 (60.0)	
Assault	0 (0.0)	2 (6.9)	1 (3.7)		1 (5.6)	0 (0.0)	
Other	1 (5.9)	0 (0.00)	5 (18.5)		0 (0.0)	0 (0.0)	
**Loss of consciousness, n (%)**				$\chi$ ^2^(2,N = 68) = 0.328, p = 0.894			$\chi$ ^2^(1,N = 21) = 0.034, p = 1.000
Yes	6 (40.0)	9 (32.1)	8 (32.0)		7 (38.9)	1 (33.3)	
No	9 (60.0)	19 (67.9)	17 (68.0)		11 (61.1)	2 (66.7)	
**Post-traumatic amnesia, n (%)**				$\chi$ ^2^(2,N = 60) = 0.198, p = 0.939			$\chi$ ^2^(1,N = 20) = 2.143, p = 0.267
Yes	4 (26.7)	7 (29.2)	7 (33.3)		6 (37.5)	0 (0.0)	
No	11 (73.3)	17 (70.8)	14 (66.7)		10 (62.5)	4 (100.0)	
**Endocrine assessment**							
**Type of dynamic testing, n (%)**							
GST	16 (94.1)	27 (93.1)	25 (96.2)		16 (88.9)	5 (100.0)	
ITT	1 (5.9)	2 (6.9)	1 (3.8)		2 (11.1)	0 (0.0)	
Peak GH (µg/L), M (SD)	1.3 (1.1)	6.1 (2.0)	16.2 (5.0)		2.2 (1.6)	1.9 (1.7)	
**Other pituitary dysfunction, n (%)**							
Yes	2 (11.8)	0 (0.0)	0 (0.0)		1 (5.6)	1 (20.0)	
No	15 (88.2)	29 (100.0)	0 (0.0)		17 (94.4)	4 (80.0)	

*CBIP* Calgary Brain Injury Program, *GH* growth hormone, *GHD* growth hormone deficiency, *GHI* growth hormone insufficiency, *GST* glucagon stimulation test, *ITT* insulin tolerance test, *mTBI* mild traumatic brain injury, *rhGH* recombinant human growth hormone.

*p < 0.05.

### Associations between clinical questionnaires and growth hormone deficiency (GHD)

**Figure 3:**
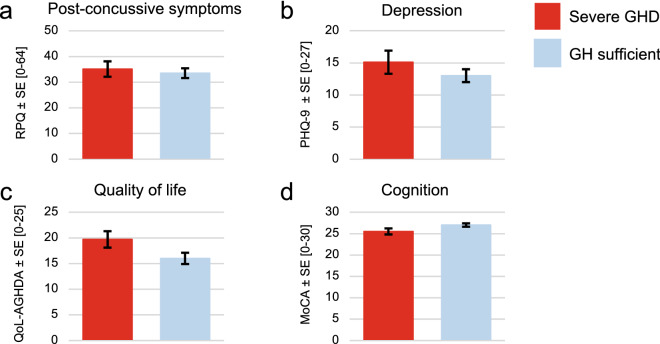
Clinical outcomes and response to dynamic testing. Participants dichotomized as severe GHD (peak GH < 3 µg/L) or GH sufficient (peak GH ≥ 3 µg/L). (**a**–**c**) Symptom burden (RPQ) (n = 48), depression (PHQ-9) (n = 60) and quality of life (QoL-AGHDA) (n = 28) scores were higher in individuals with severe GHD, however this was not statistically significant. Higher Qol-AGHDA scores indicate worse quality of life. (**d**) Cognition (MoCA) (n = 57) scores were lower in individuals with severe GHD, however this was not statistically significant. *GH* growth hormone, *GHD* growth hormone deficiency, *MoCA* Montreal Cognitive Assessment, *PHQ-9* Patient Health Questionnaire-9, *RPQ* Rivermead Post Concussion Symptoms Questionnaire, *QoL-AGHDA* Quality of Life Assessment of Growth Hormone Deficiency in Adults.

Testing for homogeneity of regression slope assumptions was performed. For each of the clinical questionnaires, interactions between the covariates and severe GHD were non-significant (p-values greater than 0.05). After adjusting for age and sex there was no association between severe GHD and measures of symptom burden (η^2^_p_ = 0.004, p = 0.658), depression (η^2^_p_ = 0.018, p = 0.311), cognition (η^2^_p_ = 0.060, p = 0.071) or quality of life using the Quality of Life Assessment of Growth Hormone Deficiency in Adults (Qol-AGHDA) (η^2^_p_ = 0.013, p = 0.067) (Fig. 3). The estimated marginal mean Montreal Cognitive Assessment (MoCA) score was 25.5 ± 0.7 for those with severe GHD. A cut-off of 26 (out of 30) is used for mild cogntive impairment.

**Figure 4:**
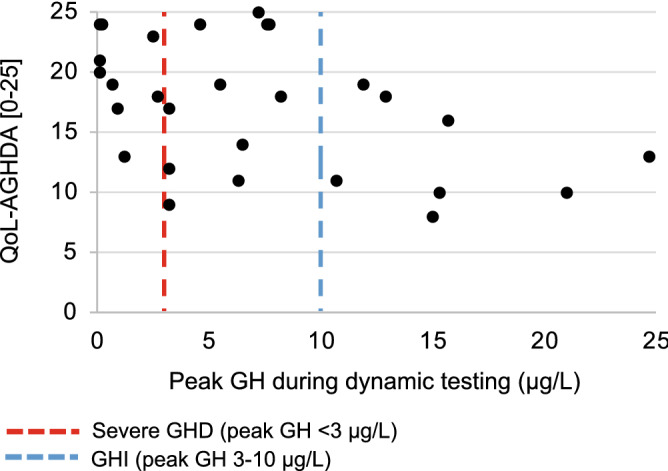
Quality of life assessment and dynamic testing results. Qol-AGHDA scores (n = 28) were correlated with peak GH during dynamic testing, r(26) = − 0.454, p = 0.015. *GH* growth hormone, *GHD* growth hormone deficiency, *GHI* growth hormone insufficiency, *QoL-AGHDA* Quality of Life Assessment of Growth Hormone Deficiency in Adults.

A subset of patients (n = 28; 38%) who underwent dynamic testing completed the Qol-AGHDA at their initial assessment by endocrinology. Compared to the full sample who received dynamic testing, those who completed the questionnaire did not differ by sex (p = 0.824), age at testing (p = 0.612) or time since injury (p = 0.881). Qol-AGHDA scores were correlated with peak GH during dynamic testing (Fig. 4).

### Growth hormone (GH) therapy

**Figure 5:**
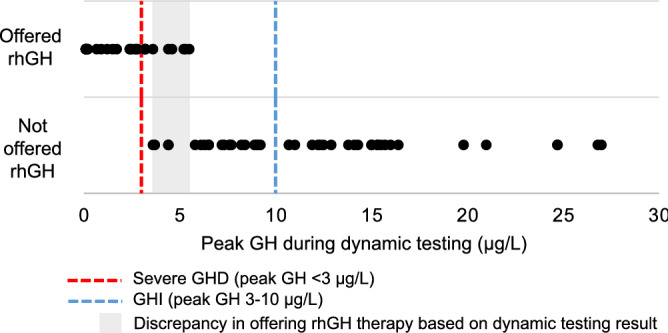
Whether rhGH therapy was offered and corresponding peak GH results. *GH* growth hormone, *GHD* growth hormone deficiency, *GHI* growth hormone insufficiency, *rhGH* recombinant human growth hormone.

Peak GH thresholds used for prescription of recombinant human GH (rhGH) therapy differed between clinicians. Twenty-six patients with a mean peak GH of 2.3 ± 1.8 µg/L (range 0.1–5.5 µg/L) were offered rhGH therapy. Of those offered therapy, 8 (31%) had a peak GH > 3 µg/L. Three patients (12%) did not proceed with rhGH due to cost or lack of insurance. Therapy was inconsistently offered for peak GH results of 3.6–5.5 µg/L. The dynamic testing results of 4 patients with peak GH result of 3.6–5.5 µg/L were interpreted as normal and therefore they were not offered rhGH (Fig. 5).

### Response

Timepoint of response determination varied depending on when patients started rhGH. Response (responder versus non-responder) was determined at their most recent clinic assessment. For 9 (39%) patients, response was determined 3 months following treatment start. Response determination for 14 (61%) patients was at 1 year or greater. At time of response determination, 18 (78%) patients had responded to treatment with self-reported benefit, deciding to continue rhGH. Of the 18 patients who responded to therapy, 13 (72%) had severe GHD and 5 (28%) had GHI. After starting rhGH, 5 (22%) patients discontinued treatment. Of those who discontinued treatment, 2 stopped after 1 year or more due to lack of continued benefit, 2 did not have self-reported benefit within the first 3 months, discontinuing rhGH and one patient reported side effects of enlarged hands and feet and soft tissue swelling.

## Discussion

It is only recently that the risk of hypopituitarism following mTBI has been recognized^14^. Many of the psychosocial symptoms of GHD overlap with persistent post-concussive symptoms suggesting that in some cases, untreated endocrinopathy may contribute to post-injury impairment^15^. Previous studies examining GHD following mTBI have focussed on combat-related^16,17^ or repetitive sport-related concussion cohorts^10^. This study presents one of the largest reported cohorts of patients assessed for GHD following mTBI. Our findings highlight the importance of screening for pituitary dysfunction in adults with persistent symptoms following mTBI, further suggesting that the majority of patients with GHD who undergo treatment improve. However, we observed great variability in the clinical management of these patients; only a small number of individuals presenting to outpatient brain injury clinic services were referred for endocrinological assessement, and even fewer progressed to GH testing and subsequent therapeutic GH replacement, possibly due to variable interpretations of GH testing. In light of the high prevalence of GHD/GHI in patients tested in this sample, and the suggested benefit of rhGH treatment, there is a great need for collaboration between specialized brain injury clinicians and endocrinologists. Further research and development of care pathways, specifically between brain injury and endocrinology services, may allow for better clinical management of those with persistent post-concussive symptoms.

Previously, post-traumatic hypopituitarism was not thought to result from milder injury. However, a systematic review of post-traumatic pituitary dysfunction reported a pooled prevalence of hypopituitarism of 16.8% following mTBI^14^. Despite this finding, consensus guidelines and expert opinions have recommended that only patients with moderate or severe TBI, an mTBI requiring hospitalization for at least 24 h, or an mTBI with an abnormality on initial CT be screened for post-traumatic pituitary dysfunction^9,18,19^. The lack of investigation of GHD following uncomplicated mTBI has likely contributed to the absence of screening recommendations for this population. Therefore, determination of which patients should be tested for GHD remains an important clinical challenge. This study provides evidence of GHD following uncomplicated mTBI, which raises questions of if and when these patients should be screened for post-traumatic hypopituitarism. Collaborative development of guidelines by brain injury and endocrinology clinicians to guide referral of patients following uncomplicated mTBI for investigation of post-traumatic GHD is an important next step.

Post-traumatic GHD may be transient, suggesting that screening acutely post-injury is not of clinical benefit^20^. However, testing in patients with persistent symptoms greater than one-year post-injury is advised as changes in pituitary function have been shown to persist at 3 and 5 years post-injury^8,21^. In this study, patients underwent dynamic testing a mean of 25 ± 11.3 months post-injury. Testing at this timepoint may be explained by several factors. Many patients did not present to the outpatient brain injury clinic less than one year post-injury and therefore may not have been referred to endocrinology sooner. Treating physicians may also elect to first try other less invasive treatment options prior to referring for assessment of pituitary dysfunction. While assessment of pituitary dysfunction at one year post-injury is appropriate as soon as referral for dynamic testing is made by the treating endocrinologist, depending on the healthcare system, wait times may determine the assessment time course. In our health care system, it may be several months from the time of referral to assessment by an endocrinologist and subsequent testing. Of those tested in our sample, 63% met criteria for severe GHD or GHI. This supports previous data that GHD can persist at one year or more following mTBI^8,21^.

Several factors may contribute to the low rate (3%) of referral of patients seen at CBIP for endocrinological assessment. Patients seen at CBIP may have moderate to severe brain injury or other acquired brain injury and therefore would not be included in this study. Additionally, many patients may present with vague clinical signs, or symptoms that may not be recognized as post-traumatic GHD. Treating physicians may also elect to pursue less invasive therapies or testing first.

Our findings suggest there is a need for better standardization of timepoints for post-traumatic pituitary dysfunction screening in this population. With an estimated prevalence of GHD of 5–40%^5–8^ at 1 year post-mTBI, it is possible that the prevalence of GHD in the total CBIP sample would be higher than reported in this study. The high treatment response rate (78%) to rhGH suggests that diagnosis and treatment of post-traumatic GHD may be especially beneficial to those presenting to outpatient brain injury clinics with persistent symptoms. However, there are currently no specific guidelines or screening tools that can aid clinicians in determining who should be referred for screening of post-traumatic GHD. Given that dynamic testing is labor intensive and expensive, it is not feasible to test all patients with mTBI and persistent symptoms one year post-injury. There is a need for a more cost-effective screening measure for patients with persistent symptoms following uncomplicated mTBI. There is also a need for a comprehensive prospective study evaluating reason for referral for assessment of pituitary dysfunction following mTBI by brain injury clinicians.

Dynamic testing is required for assessment of GHD as serum IGF-1 lacks diagnostic sensitivity and is not recommended by clinical practice guidelines^22^. In a mixed-severity traumatic brain injury cohort, of the 25 individuals diagnosed with GHD based on dynamic testing, IGF-1 level was within the age and gender specific range for all patients, confirming lack of diagnostic utility of IGF-1 in the diagnosis of GHD in this patient population^23^. Despite this, 3 patients referred to endocrinology had serum IGF-1 as the only assessment of GHD. As the remainder of patients received dynamic testing for assessment of GHD, IGF-1 data are not available for the majority of this cohort and thus comparison between dynamic testing results and IGF-1 was not possible. Unfortuantely, in some cases, recent studies have still used IGF-1 as a measure of GHD^16^.

Interpretation of post-traumatic GHD literature is also complicated by the use of various dynamic tests. The ITT is often cited as the gold-standard, while GST is frequently used when ITT is contra-indicated. GHRH + Arginine and GHRH + AGRP-6 tests have also been used, although these are no longer available in Canada. The majority of this sample underwent GST (94%) for assessment of GHD. Additionally, diagnostic cut off values vary across groups and may or may not be body mass index (BMI)-adjusted. The most recent American Association of Clinical Endocrinologists (AACE) guidelines suggest a normal response to ITT is indicated by a peak GH > 5 µg/L, while a normal response to GST should be > 3 µg/L for BMI < 25 kg/m^2^ and > 1 µg/L for BMI > 25 kg/m^2^^22^. Few studies have used BMI-adjusted interpretation of GST data and a variety of other cut-offs are used in the literature. A recent randomized control trial of rhGH for treatment of GHD following mTBI used a broader diagnostic criteria, including individuals with a peak GH < 8 µg/L during GST^11^. Using this criteria, 14 additional patients in this sample would have met criteria for rhGH treatment.

We observed a wide variation in the interpretation of test results and decisions to offer rhGH replacement therapy among endocrinologists. This may reflect the variation in biochemical thresholds for diagnosis recommended by different guidelines. As well, most studies of GHD in the endocrinology literature are conducted in patients with pituitary surgery. Therefore, many endocrinologists may be unware or uncertain as to whether mTBI patients should be diagnosed with GHD following the same guidelines. Lastly, there are very few studies of rhGH replacement in those with mTBI such that endocrinologists may be reluctant to offer rhGH therapy due to uncertainty of benefit. Our findings highlight the benefit of treating individuals with GHD following mTBI. A large randomized controlled trial evaluating the efficacy and benefits of GH replacement is needed. There is also a great need for determination of response according to peak GH during dynamic testing to inform implementation of a standardized cut-off point for treatment of these patients.

Few clinical determinants of GHD following mTBI have been identified. Previous literature has reported associations between post-traumatic GHD and older age, increased BMI and waist circumference^6,17,24–26^. Injury severity based on GCS has not been found to be associated with GHD^27,28^. In this study, we found that none of the clinical measures commonly collected in the outpatient brain injury clinic setting are significantly associated with severe GHD, although this may be due in part to the relatively small sample size. A larger, adequately powered study is needed to evaluate clinical predictors of GHD in individuals with uncomplicated mTBI.

We did not find a significant difference in cognitive impairment between individuals with and without severe GHD. While not significant, the estimated marginal mean MoCA score in those with GHD met criteria for mild cognitive impairment, while the MoCA score for those without GHD did not. Similarly, decreased inhibition of cognitive interference has been reported in veterans with GHD following combat-related mTBI^17^. The measures used in these studies cannot be directly compared and the study samples were notably different (civilians versus veterans). Associations between cognition and GHD have primarily been reported in cohorts following moderate or severe TBI. Several studies have reported lower scores on measures of attention and memory in those with GHD^26,28^. Additionally, lower scores on the Level of Cognitive Functioning Scale at hospital discharge were positively correlated with peak GH levels (during dynamic testing) in a sample 6–12 months following moderate or severe TBI^29^. In non-TBI individuals with GHD, cognitive performance was shown to improve with GH replacement in a meta-analysis of 340 patients^30^. Future work should assess cognition in individuals with and without GHD following mTBI using a full neuropsychological battery to examine if a specific sub-test(s) may be a useful preliminary screen of hypopituitarism. Establishing one or multiple clinical measures that are most predictive of post-traumatic GHD would help to inform screening guidelines and be of great clinical utility.

Although not found in this sample, depression has been associated with GHD in other cohorts. Higher depression scores using the Beck Depression Inventory were reported in those with GHD following combat-related mTBI^17^. Higher incidence of depression has also been reported in mixed-severity cohorts using a variety of measures^15,31^. Again, the discrepancy in findings is likely due to differences in injury severity, mechanism of injury and outcome measures between cohorts. Our findings suggest there is a high prevalence of depression in those with persistent post-concussive symptoms presenting to outpatient brain injury services, and therefore depression alone this is not specifically related to post-traumatic GHD.

A correlation with medium effect size was previously reported between severe GHD and Qol-AGHDA scores following combat-related mTBI^17^. Similarly, we reported a correlation between peak GH and Qol-AGHDA scores. While this measure should not be used diagnostically, it may be a superior measure for initial screening of GHD in the mTBI population, compared to specific symptom questionnaires. In a cohort following moderate-severe TBI, scores on the Qol-AGHDA were not significantly different between those with and without GHD. However, the analysis included individuals with both GHD and GHI^15^. On the Short Form (36) Health Survey, a quality of life measure, decreased scores in domains of energy and fatigue and emotional well-being have been reported in two separate mixed-severity TBI cohorts with GHD^15,31^. Our findings support previous work, demonstrating reduced quality of life in those with GHD following TBI and highlights the importance of screening and diagnosis of GHD in these patients.

Of those offered therapy, 88% started rhGH. This illustrates the willingness of patients to follow injection protocols and highlights the effect of persistent symptoms on quality of life and function in this cohort. The benefits of rhGH for GHD have been studied following moderate and severe TBI^12,13^, but few studies have evaluated the response to treatment following mTBI^11^.

The literature is unclear on whether or not GH therapy improves cognition. In a placebo-controlled crossover study of individuals with GHD following mTBI, there was not a significant improvement on neuropsychological testing measures following GH replacement^11^. However, in a sample of individuals with moderate or severe injury, several measures on a neuropsychological battery did improve following a year of GH replacement^12^. While improvements in cognition were not reported with GH replacement in those with mTBI, treatment lead to improvements in fatigue, sleep, mood and an increase in lean body mass^11^. In our sample, 78% of patients who started GH therapy had self-reported benefit and decided to continue with treatment. Unfortunately, lack of testing resources, specialized clinicians and treatment cost may all limit accessibility to treatment. In this cohort, three patients did not proceed with treatment due to associated cost or lack of insurance coverage.

There is evidence to suggest that greater improvement with rhGH treatment may occur over time, specifically in the first year^12^. Therefore, we suspect that if individuals reported improvement of symptoms at 3 months, the response would be even more pronounced at 1 year. Interestingly, two patients decided to stop GH replacement following 1 year or more of therapy. Following prescription of rhGH, current practice is to follow-up with patients at 3 months and yearly thereafter. Response to intervention is assessed at follow-up visits by self-reported benefit and willingness to continue therapy, as described in this paper. It may be of interest to administer common clinical outcomes for objective assessment of symptom improvement over time. The timecourse of response to rhGH in those with post-traumatic GHD is not well understood and further studies are needed to better understand response over time.

This study had several limitations. This cohort is not representative of all adults with mTBI, but rather individuals with months to years of symptoms following injury. Although this was a specific patient population, the results may be generalizable to patients with persistent post-concussive symptoms greater than 1 year. The variable time since injury at clinic assessment and dynamic testing in addition to variable timepoint of response determination are limitations. While we did not have access to BMI data, it is an important factor in the interpretation of dynamic testing results. Future studies with consistent timepoints for testing and treatment response in individuals with mTBI are required.

Additional studies are needed to establish the prevalence of GHD following mTBI. This study only included patients specifically referred for assessment of post-traumatic hypopituitarism and therefore is not indicative of overall prevalence. To date, prevalence data is based on small cohorts following complicated or combat-related mTBI. There is also a great need to identify risk factors of post-traumatic GHD following mTBI. This would serve to inform referral for testing and enable efficient allocation of testing resources. Additional studies are needed to systematically evaluate change in clinical outcome measures (i.e., cognition, quality of life, post-concussive symptoms) following rhGH treatment across timepoints (i.e., 6, 12 months).

## Conclusion

Hypopituitarism, specifically GHD, is likely more common in individuals following mTBI than previously appreciated. Treatment for individuals with persistent post-concussive symptoms is largely symptom-based and symptom etiology is often difficult to establish. Our findings provide further evidence that pituitary dysfunction following mTBI should not be ignored and may contribute to chronic symptoms. However, lack of screening tools and specific guidelines to aid clinicians in determining which patients should be screened following uncomplicated mTBI likely contributes to the great variability in screening of these patients. Post-traumatic GHD is a serious, but treatable complication of mTBI. This study demonstrates the need for consensus on referral for testing, interpretation of dynamic testing results and offering of rhGH treatment. Currently the clinical management of these patients is varied and there is no evidence-based gold standard diagnostic cut-off for offering rhGH based on dynamic testing results for post-traumatic GHD. Many patients with post-traumatic GHI, generally defined as a peak GH of 3-10 µg/L, may also benefit from rhGH therapy, and the use of narrow diagnostic criteria should be reconsidered for this patient population.

## Methods

### Participants

We performed a retrospective chart review of patients seen in both CBIP and OECs between September 2016 and 2019. Patient charts were included in the review based on the following: (1) referral from CBIP to an OEC for investigation of post-traumatic pituitary dysfunction (referral at the discretion of the treating physician); (2) diagnosis of mTBI based on American Congress of Rehabilitation Medicine criteria^32^ and persistent post-concussive symptoms. Individuals with moderate or severe TBI or other acquired brain injury were excluded. Relevant demographic, injury characteristic and past medical history information was extracted from electronic medical records. Demographic information included age, sex, handedness, education and employment status. Injury characteristics included time since injury, mechanism of injury, and whether or not there was loss of consciousness or post-traumatic amnesia. Past medical history of endocrine disorder or previous mTBI was noted.

### Ethics approval

This study was approved by the Conjoint Health Research Ethics Board at the University of Calgary (REB19-0663) and as such, performed in accordance with the relevant guidelines and regulations. Informed consent was waived by the Conjoint Health Research Ethics Board at the University of Calgary.

### Data collection

The following clinical questionnaire data collected at patients’ initial brain injury clinic assessment was extracted.

1. The Rivermead Post Concussion Symptoms Questionnaire (RPQ) is a commonly used questionnaire where 16 post-concussion symptoms are scored between 0 (not experienced at all) and 4 (a severe problem)^33^. A score out of 64 is generated.
2. The Patient Health Questionnaire-9 (PHQ-9) is a brief measure of depressive symptoms^34^. This 9-item measure (scored out of 27) is a valid assessment of depression following TBI^35^.
3. The MoCA is a brief measure of cognitive impairment scored out of 30^36^.

A subset of patients completed the Qol-AGHDA at their initial endocrinology clinic assessment. This is a 25-item assessment of quality of life designed as a preliminary screening tool for GHD where higher scores indicate worse quality of life^20^.

Investigations of GHD ordered by treating endocrinologists were collected, including whether or not an ITT or GST was performed. The decision to proceed with GHD testing, test result interpretation and decision to offer GH replacement were at the discretion of the attending endocrinologist. Presence of other pituitary dysfunction, such as hypogonadism, was also collected. Completion of ITT versus GST was based on availability of nursing staff or contraindication to ITT such as severe cardiovascular disease. All dynamic GH testing (GST or ITT) was completed at testing centers under the supervision of a specialized nurse and followed standard published protocols^37^. Results of dynamic testing were recorded. In accordance with clinical guidelines, peak stimulated GH levels < 3 µg/L were considered severe GHD^38^. GH insufficiency (GHI) was indicated by a peak stimulated GH of 3–10 µg/L, while a response > 10 µg/L was considered normal.

Patients seen by an endocrinologist who did not undergo dynamic testing were classified from electronic medical records as follows: (1) patient reported symptom improvement; (2) patient declined testing; (3) endocrinologist did not order testing/determined it was not indicated; (4) unknown.

Number of patients who started rhGH therapy was collected. Treatment was offered based on the clinical expertise of the treating endocrinologist. For patients offered rhGH who did not proceed with treatment, reasons for declining were recorded. Patients who started rhGH were dichotomized as either treatment responders or non-responders in accordance with our previously published approach whereby patients self-determine the overall value of GH replacement^39^. An individual was classified as a responder if they had self-reported symptom improvement and decided to continue therapy at their most recent clinic visit. Response determination timepoints were at 3 months following treatment start, or greater than one year. Individuals who had started but elected to discontinue rhGH therapy were classified as non-responders.

### Statistical analysis

Participant demographics, past medical history, injury characteristics and endocrinology assessment data were reported. Cut-offs based on results of dynamic testing were used for classification of GHD. Between group differences were analysed using either chi square or independent-samples t-tests where appropriate. A sensitivity analysis for GHD prevalence was performed using different peak serum GH cut-offs (during dynamic testing) commonly used in the literature for diagnosis of GHD, while also accounting for assay uncertainty (± 0.3 µg/L)^4,11^. Therefore, cut-offs of < 1.0 µg/L, < 1.3 µg/L, < 3.0 µg/L, < 3.3 µg/L, < 5.0 µg/L, < 5.3 µg/L, < 8.0 µg/L and < 8.3 µg/L were included in the analysis. Differences in clinical outcomes between those with and without severe GHD were compared using one-way analysis of covariance (ANCOVA), controlling for age and sex as these may influence GH levels. Association between QoL-AGHDA scores and peak GH on dynamic testing was analyzed using Pearson’s correlation.

## Data Availability

The data from this study are available upon request.
